# Polysaccharide-Based Materials: Developments and Properties

**DOI:** 10.3390/polym17223028

**Published:** 2025-11-14

**Authors:** Andrés Gerardo Salvay

**Affiliations:** Laboratorio de Obtención, Modificación, Caracterización y Evaluación de Materiales, Departamento de Ciencia y Tecnología, Universidad Nacional de Quilmes, Roque Sáenz Peña 352, Bernal B1876BXD, Argentina; asalvay@unq.edu.ar

The growing concern for environmental sustainability and the urgent demand to reduce dependence on non-renewable resources have placed bio-based materials at the centre of materials science innovation. Within this context, polysaccharides have emerged as one of the most promising classes of biopolymers. Their natural abundance, structural versatility, chemical functionality, and intrinsic biodegradability make them ideal candidates for advanced, functional, and sustainable materials [1,2].

Polysaccharides are among the most abundant biopolymers on Earth, sourced from plants, marine organisms, and microorganisms [3]. Their extraction and utilisation represent an essential step towards a circular bioeconomy. The structural complexity of polysaccharides, arising from their monosaccharide composition, linkage types, branching patterns, and degrees of substitution, provides a rich foundation for tailoring their material properties [4]. This versatility enables their conversion into a wide variety of functional forms such as hydrogels [1], films [2], aerogels [5], and composites [6,7].

In recent years, increasing attention has been paid to the valorisation of agricultural and industrial by-products as sources of polysaccharides [8]. At the same time, microbial fermentation has gained relevance as a sustainable alternative route, capable of producing exopolysaccharides (EPS) with well-defined structures and properties [9]. Microbial EPS such as dextran, pullulan, gellan, and bacterial cellulose have been extensively investigated as renewable matrices for high-performance materials [10].

The conversion of polysaccharides into materials with targeted performance involves a complex interplay between molecular structure, processing strategy, and application requirements. Physical treatments, chemical modifications, crosslinking reactions, and blending with other biopolymers or plasticisers are often used to overcome intrinsic limitations such as brittleness, hydrophilicity, or poor water barrier properties [11,12]. Hybridization with nanomaterials has proven effective to reinforce polysaccharide matrices [13]. Crosslinking reactions, either covalent or non-covalent, have proven particularly effective in enhancing hydrogels’ stability and functional performance [14]. From a biotechnological viewpoint, enzyme-mediated transformations and green synthesis routes are increasingly preferred over conventional chemical treatments, offering more environmentally benign processes [15].

The versatility of polysaccharide-based materials translates into an impressive range of applications. In biomedicine, polysaccharide hydrogels and films have been developed as carriers for controlled drug delivery, scaffolds for tissue regeneration, and wound-healing dressings [16]. Their intrinsic biocompatibility, non-toxicity, and tunable degradation profiles make them attractive for applications requiring close interaction with biological tissues. In the food and packaging industries, polysaccharide films and coatings serve as biodegradable alternatives to petroleum-based plastics, offering tunable permeability and mechanical properties [2]. Incorporation of natural antimicrobials or antioxidants into such films has led to the emergence of active packaging materials that can extend the shelf life of food products [7]. In environmental technologies, polysaccharide-based adsorbents and membranes are increasingly used for wastewater treatment and pollutant removal, demonstrating excellent performance due to their high surface area and functional group diversity [17]. Furthermore, the integration of polysaccharides into hybrid and composite materials opens new possibilities for advanced applications in sensors and flexible electronics [18].

Despite significant advances, key challenges remain before polysaccharide-based materials can achieve their full potential. Mechanical robustness, high hydrophilicity, and variability in properties arising from differences in natural sources or extraction methods continue to hinder large-scale adoption. Addressing these issues requires both improved formulation and processing techniques and the standardisation of feedstock characterisation. The design of hybrid materials, combining polysaccharides with other biopolymers, nanofillers, or synthetic polymers, represents one of the most promising strategies to overcome current limitations. Likewise, nanostructuration and self-assembly strategies offer innovative routes to control morphology and enhance performance at multiple scales. Computational modelling and molecular simulations are also expected to play an increasingly important role in predicting and optimising material behaviour. Moreover, true sustainability cannot be achieved without considering the entire lifecycle of materials. Future efforts should integrate environmental and economic assessments, ensuring that bio-based materials deliver genuine ecological advantages compared with conventional counterparts.

In this Special Issue, we have collected the most recent advances in developments and properties of polysaccharide-based materials, including ten original research articles. The research topics mainly cover the design of novel polysaccharide-based materials and composites, the valorisation of bio-based resources, and the exploration of functional properties for applications.

Polysaccharides are increasingly exploited as structural platforms for advanced functional materials. Kadsanit et al. [19] investigated the synthesis and modification of dialdehyde bacterial cellulose (DBC) through controlled periodate oxidation. Using response surface methodology, the authors established predictive correlations between the reaction conditions and the degree of oxidation, which strongly influenced morphology and aldehyde content. The resulting DBC acted as an efficient crosslinking and reinforcing agent for gelatine matrices, enhancing tensile strength and stability. Moreover, the ability to fine-tune the oxidation degree provides additional flexibility in designing materials with tailored sponge-like morphologies and mechanical properties suitable for biomedical applications. Li et al. [20] reported a sustainable strategy for developing high-performance ionic conductive flexible hydrogels from Camellia oleifera shells, an agricultural by-product. By extracting nanocellulose and introducing zwitterionic functionalities via atom transfer radical polymerisation, they fabricated nanocellulose–polyvinyl alcohol (NC-PSBMA/PVA) composite hydrogels with remarkable porosity, conductivity, and mechanical strength seven times greater than neat PVA. The resulting materials exhibit high sensitivity and rapid response, representing a promising route towards biodegradable and high-efficiency flexible electronic sensors.

In the field of energy storage, Legerstee et al. [21] examined magnesium transfer phenomena in aqueous alginate-based electrolytes using atomic force microscopy. Their findings demonstrated reversible magnesium deposition and stripping without dendrite formation, enabling safe and controlled operation. This work underscores the potential of polysaccharide-based electrolytes to improve the stability and environmental compatibility of emerging magnesium battery technologies.

Several contributions focused on circular-economy approaches and eco-friendly solutions derived from polysaccharide-rich wastes. Lamoudan et al. [22] proposed a pioneering method to transform cellulose-containing textile waste into multifunctional construction panels using a papermaking process. The integration of phosphorylated lignocellulosic fibres endowed the panels with enhanced mechanical resistance, thermal insulation, and fire-retardant properties. Meeting international construction standards, these panels exemplify how upcycling textile residues can simultaneously reduce waste and yield value-added materials for sustainable architecture. Complementing this perspective, Trivunac et al. [23] converted discarded cotton and mixed yarns into high-performance carbon adsorbents through hydrothermal carbonisation and KOH activation. The modified cellulose-based materials displayed large surface areas and abundant oxygen functionalities, leading to high adsorption capacity for heavy metals, especially lead. This approach not only valorises textile waste but also provides a scalable solution for wastewater treatment, reinforcing the environmental potential of polysaccharide-based sorbents.

Wang et al. [24] investigated chitosan as a green alternative to cement for soil stabilisation. Their systematic evaluation revealed that optimal acid concentration (0.5–1%) and curing temperature (45–65 °C) significantly enhance unconfined compression strength and durability. Microscopic analyses confirmed that chitosan forms hydrogen and electrostatic interactions with soil particles, filling voids and strengthening the matrix. The results establish chitosan as a sustainable and effective soil-binding agent for eco-friendly civil engineering.

Hydrogels, films, and coatings based on polysaccharides continue to attract attention for their biocompatibility and tunable properties. Gorroñogoitia et al. [25] designed semi-interpenetrating (semi-IPN) hydrogels combining alginate and sulphated hyaluronic acid for articular cartilage tissue engineering. By adjusting the alginate-to-hyaluronic acid ratio, the researchers achieved control over rheological and viscoelastic behaviour, optimising the balance between mechanical performance and biological functionality. These hydrogels effectively mimic extracellular matrix properties, demonstrating strong potential for regenerative medicine.

In line with food packaging and food preservation, Nitikornwarakul et al. [26] studied glutinous rice starch–chitosan (GRS/CS) blends as active coatings to extend mango shelf life. Increasing chitosan content enhanced mechanical strength, hydrophobicity, and resistance to fungal infection, maintaining fruit quality for up to ten days compared to two days for uncoated samples. The optimised coatings reduced dehydration and respiration, confirming their applicability as biodegradable, natural-based films for post-harvest preservation. Shlosman et al. [27] developed cellulose-encapsulated emulsions of thymol and eugenol essential oils through lyophilisation. The resulting powders retained antimicrobial and antioxidant properties, showed high encapsulation efficiency, and exhibited slow release and improved thermal stability. These characteristics make them suitable for integration into polymer matrices, particularly for agricultural and packaging applications requiring controlled release at elevated temperatures.

Further extending the exploration of microbial polysaccharides, Ramírez Tapias et al. [28] investigated films derived from integral milk kefir grain biomass and purified kefiran. Both materials formed homogeneous structures, yet kefiran films were more transparent and exhibited stronger polymeric interactions. The addition of glycerol acted as a plasticiser in integral milk kefir grain biomass-based films. On the other hand, an antiplasticisation effect at low glycerol levels in purified kefiran films points to complex polymer–plasticiser interactions. These findings emphasise the differences between materials derived from the integral milk kefir grains and those from purified kefiran, providing insights into their application potential.

The ten contributions collected in this Special Issue illustrate the remarkable versatility of polysaccharides as sustainable, functional building blocks for advanced materials. Through innovative processing, controlled chemical modification, and synergistic blending with other biopolymers, these studies reveal how cellulose, chitosan, alginate, hyaluronic acid, starch, and kefiran can yield materials with tunable mechanical, electrical, and biological properties. Applications range from flexible sensors and biomedical hydrogels to sustainable packaging, wastewater treatment, and soil stabilisation. Collectively, they demonstrate that polysaccharide-based systems can effectively bridge the gap between renewable resource utilisation and high-performance engineering solutions.

## References

[1] Berradi A., Aziz F., El Achaby M., Ouazzani N., Mandi L.A. (2023). Comprehensive review of polysaccharide-based hydrogels as promising biomaterials. Polymers.

[2] Goyal R., Verma S., Vaish M., Shekhar A. (2025). Polysaccharide: A biodegradable biopolymer for edible films and coating. J. Current Res. Food Sci..

[3] Benalaya I., Alves G., Lopes J., Silva L.R. (2024). A review of natural polysaccharides: Sources, characteristics, properties, food, and pharmaceutical applications. Int. J. Mol. Sci..

[4] Xu B.-W., Li S.-S., Ding W.-L., Zhang C., Rehman M.U., Tareen M.F., Wang L., Huang S.-C. (2025). From structure to function: A comprehensive overview of polysaccharide roles and applications. Food Front..

[5] Muhammad A., Lee D., Shin Y., Park J. (2021). Recent progress in polysaccharide aerogels: Their synthesis, application, and future outlook. Polymers.

[6] Lago A., Delgado J.F., Rezzani G.D., Cottet C., Ramírez Tapias Y.A., Peltzer M.A., Salvay A.G. (2023). Multi-component biodegradable materials based on water kefir grains and yeast biomasses: Effect of the mixing ratio on the properties of the films. Polymers.

[7] Li S., Ren Y., Hou Y., Zhan Q., Jin P., Zheng Y., Wu Z. (2024). Polysaccharide-based composite films: Promising biodegradable food packaging materials. Foods.

[8] Maqsood S., Khalid W., Kumar P., Benmebarek I.E., Rasool I.F.U., Trif M., Moreno M., Esatbeyoglu T. (2025). Valorization of plant-based agro-industrial waste and by-products for the production of polysaccharides: Towards a more circular economy. Appl. Food Res..

[9] Mouro C., Gomes A.P., Gouveia I.C. (2024). Microbial exopolysaccharides: Structure, diversity, applications, and future frontiers in sustainable functional materials. Polysaccharides.

[10] Coma M.E., Peltzer M.A., Delgado J.F., Salvay A.G. (2019). Water kefir grains as an innovative source of materials: Study of plasticiser content on film properties. Eur. Polym. J..

[11] Liu T., Ren Q., Wang S., Gao J., Shen C., Zhang S., Wang Y., Guan F. (2023). Chemical modification of polysaccharides: A review of synthetic approaches, biological activity and the structure–activity relationship. Molecules.

[12] Janik W., Jakubski L., Kudła S., Dudek G. (2024). Modified polysaccharides for food packaging applications: A review. Int. J. Biol. Macromol..

[13] Trache D., Thakur V.K., Boukherroub R. (2020). Cellulose nanocrystals/graphene hybrids—A promising new class of materials for advanced applications. Nanomaterials.

[14] Zhang R., Liu X., Zhang W., Cui B., Du Y., Huang Y., Li W., Liu Q., Ren C., Tang Z. (2025). A review of polysaccharide-based hydrogels: From structural modification to biomedical applications. Int. J. Biol. Macromol..

[15] Schmid J., Sieber V. (2015). Enzymatic transformations involved in the biosynthesis of microbial exo-polysaccharides based on the assembly of repeat units. ChemBioChem.

[16] Ma C., Du L., Guo Y., Yang X. (2024). A review of polysaccharide hydrogels as materials for skin repair and wound dressing: Construction, functionalization and challenges. Int. J. Biol. Macromol..

[17] Díaz Bukvic G., Rossi E., Errea M.I. (2023). Polysaccharides as economic and sustainable raw materials for the preparation of adsorbents for water treatment. Polysaccharides.

[18] Wang Y., Huang Z., Zhang D., Li M., Wen X., Lei X., Ye Z., Pang J. (2025). High performance multifunctional bio-gel sensor prepared with konjac glucomannan and κ-carrageenan. Carbohydr. Polym..

[19] Kadsanit K., Sriariyanun M., Phisalaphong M., Kirdponpattara S. (2025). Tailoring dialdehyde bacterial cellulose synthesis for versatile applications. Polymers.

[20] Li J., Peng W., Lei Z., Jian J., Cong J., Zhao C., Wu Y., Su J., Han S. (2025). Fabrication of zwitterionized nanocellulose/polyvinyl alcohol composite hydrogels derived from camellia oleifera shells for high-performance flexible sensing. Polymers.

[21] Legerstee W.J., Kiriinya L., Kwakernaak M., Kelder E.M. (2024). Magnesium transfer between atomic force microscopy probes and metal electrodes in aqueous alginate electrolytes. Polymers.

[22] Lamoudan H., Abenghal L., Belosinschi D., Brouillette F., Dolez P., Panneton R., Fonrouge C. (2024). Sustainable transformation of cellulose-containing textile waste into multifunctional panels with tailored fr-lignocellulosic fibres. Polymers.

[23] Trivunac K., Mihajlović S., Vukčević M., Maletić M., Pejić B., Kalijadis A., Perić Grujić A. (2024). Modified cellulose-based waste for enhanced adsorption of selected heavy metals from wastewater. Polymers.

[24] Wang R., Ong D.E.L., Sadighi H., Goli M., Xia P., Fatehi H., Yao T. (2025). Optimizing soil stabilization with chitosan: Investigating acid concentration, temperature, and long-term strength. Polymers.

[25] Gorroñogoitia I., Olza S., Alonso-Varona A., Zaldua A.M. (2025). The effect of alginate/hyaluronic acid proportion on semi-interpenetrating hydrogel properties for articular cartilage tissue engineering. Polymers.

[26] Nitikornwarakul C., Wangpradid R., Rakkapao N. (2024). Impact of molar composition on the functional properties of glutinous rice starch–chitosan blend: Natural-based active coating for extending mango shelf life. Polymers.

[27] Shlosman K., Rein D.M., Shemesh R., Cohen Y. (2024). Lyophilized emulsions of thymol and eugenol essential oils encapsulated in cellulose. Polymers.

[28] Ramírez Tapias Y.A., Rezzani G.D., Delgado J.F., Peltzer M.A., Salvay A.G. (2024). New Materials from the Integral Milk kefir grain biomass and the purified kefiran: The role of glycerol content on the film’s properties. Polymers.

